# Acceptance of HPV vaccination in boys among mothers from selected churches in Accra, Ghana

**DOI:** 10.1186/s12889-023-16028-5

**Published:** 2023-06-01

**Authors:** Evans Osei Appiah, Ezekiel Oti-Boadi, Stella Appiah, Mohammed Ali Bakkari, Manuela Akosua Menka, Dorothy Baffour Awuah, Samuel Kontoh, Awube Menlah, Isabella Garti, Susana Agyekum Boateng

**Affiliations:** 1 School of Nursing and Midwifery, Department of Midwifery, Valley View University, P.O. Box DT 595, Oyibi, Ghana; 2 Purdue University, West Lafayette, USA; 3 School of Nursing and Midwifery, Valley View University, Oyibi, Ghana; 4 Department of Nursing, Valley View University, Box AF 595, Adentan, Accra Ghana; 5 Department of pharmaceutics, College of Pharmacy, Jazan University, Jazan, Saudi Arabia; 6 Valley View University, Oyibi, Ghana; 7 University of Charles Darwin, Darwin, Australia

**Keywords:** Acceptance, HPV, Boys, Churches, Mothers, Vaccination

## Abstract

**Background:**

Almost all cases of cervical and anal cancer have been linked to the human papillomavirus (HPV). However, in addition to women who develop HPV-related cervical cancer, both men and women can also develop cancers of the anus, oral cavity, and oropharynx that are attributed to HPV. However, literature on HPV vaccination among boys globally, in Africa, and most especially in Ghana is scarce. Thus, the main objective of this study was to explore the acceptance of HPV vaccination in boys among mothers from selected churches in Accra, Ghana.

**Methods:**

In this study, a qualitative exploratory design was utilized to enlist 30 mothers who have male children aged between 9 and 12 years from the Greater Accra Region of Ghana. The recruitment of participants was carried out using a purposive sampling technique, and they were subsequently interviewed in-depth in a face-to-face setting, with the entire conversation being recorded for reference. After transcription, the recorded data were analyzed through content analysis.

**Findings:**

Upon analyzing the data, two (2) primary themes and 11 sub-themes emerged. The research showed that although the majority of the mothers were unaware of HPV in boys, they perceived it as a positive initiative and expressed a willingness to allow their sons to receive the vaccine. However, some participants mentioned certain factors that they believed could hinder the acceptance of HPV vaccination in boys among mothers. These included concerns about injection-related pain, high cost, and fears that the vaccine could make men immoral or infertile.

**Conclusion:**

The study revealed poor awareness of HPV vaccination in boys among mothers, and hence, suggested the need to increase the awareness on HPV vaccination in boys among mothers as well as the public to increase its acceptance.

## Introduction

HPV has been linked to nearly all cases of cervical and anal cancer and was responsible for 43,371 cases of cancer in 2015 [1]. Recently, a 2021 study identified that HPV-positive invasive cervical cancer was 651 per 1000 cases [2]. In 2022, a systematic review conducted identified that approximately 657 317 cancers are associated with HPV, and out of these, 264 019 (40.2%) developed in men and 393 298 (59.8%) in women [3]. Cervical cancer alone resulted in more than half (206 075 [52.4%]) of the HPV cancers [3]. Cancer of the cervix is a slow-growing cancerous disease that takes so many years to undergo malignant transformation and is associated with papillomavirus (HPV) [4]. Dyne and colleagues added that it takes 10–15 years to develop. Globally, an estimated 527,624 new cases and 265,672 deaths are recorded annually due to cervical cancer [4]. Consequentially, cancer of the cervix is ranked as the third most common cancer among women across the globe and has been regarded as a major public health issue [5].

Sub-Saharan Africa has the highest incidence and mortality rates of cervical cancer, with an annual diagnosis of 34.8 new cases per 100,000 women and 22.5 deaths per 100,000 [6]. In Ghana, cervical cancer is the second most common cancer, causing more than 3000 new cases and 2000 deaths annually. It also contributes to almost 10% of all cancer-related deaths among women in the country [6, 7]. Titiloye et al. in Nigeria reviewed 1,094 cervical histological findings and found that 1,087 of them (99.4%) were cervical carcinomas [8]. Recently Nartey et al. reported that the actual incidence and mortality rates for the general population are unknown due to an absence of a national-based population cancer registry [9].

Incorporating boys into a girls-only vaccination program, which has already demonstrated efficacy in United Kingdom, exhibits minimal cost-effectiveness, owing to the high level of protection that males receive through herd immunity. For example, all threshold prices escalate when utilizing a discount rate of 1.5%, and the addition of boys to the program becomes financially feasible, ranging from £36 to £47 [10]. In addition, a study conducted in Spain has identified that a gender-neutral 9-valent HPV vaccination for both genders is more cost-effective and beneficial. This is due to the additional protection it offers against penile cancer and oropharyngeal cancers [11]. Furthermore, in Australia, it was ascertained that most of the mothers were in support of school-based HPV vaccination for young boys and girls [12].

The primary approach for preventing cervical cancer involves vaccinating both males and females against human papillomavirus (HPV), while secondary prevention concentrates on screening and treating women who are 30 years and older [13]. Developed countries have observed a significant decline in cervical cancer cases by 65% over a span of 40 years through HPV vaccination and screening programs [14]. Furthermore, several cervical cancer cases can be prevented by vaccinating young boys and girls against HPV 16 and HPV 18 which cause about 70% of cervical cancers.

HPV infection can lead to cancers of the anus, oral cavity, and oropharynx in men, as well as genital warts, resulting in an equivalent burden for both genders [15]. It is noteworthy that all men, irrespective of their sexual orientation, carry a significant burden of HPV-associated infections [16, 17]. A significant rise in the burden of HPV-related diseases has been observed in developed countries. However, the vaccination of boys can substantially reduce this burden. By including boys in the vaccination program, more comprehensive herd immunity can be achieved, resulting in a notable decrease in viral load among the general population [18].

Over the years, numerous studies have been conducted in various countries across sub-Saharan Africa (SSA) to investigate human papillomavirus (HPV) vaccination in adolescent females [19, 20]. In Ghana, there has been a considerable amount of research on cervical cancer screening and HPV vaccination in women, which has provided valuable insights and knowledge in this area [21–23]. However, despite the growing body of literature on HPV vaccination, research on its administration to boys in Africa, especially in Ghana, remains scarce. Therefore, the current study aims to bridge this knowledge gap and investigate the views of mothers regarding the acceptance of HPV vaccination for young boys in selected churches in Accra, Ghana. This study represents one of the first attempts to explore the attitudes of mothers toward HPV vaccination for boys in Ghana. By doing so, we hope to contribute to the understanding of how HPV vaccination is perceived and accepted among the Ghanaian population.

The Health Belief Model (HBM) is the theoretical framework that best suits our study. Developed in the 1950s, the model is widely used to study the behavior of individuals regarding healthcare and disease prevention [24, 25]. The HBM assumes that people’s beliefs and perceptions about health risks, as well as the benefits and barriers of a particular behavior, influence their decision-making process. The HBM comprises several constructs that help to explain the decision-making process of individuals, including perceived susceptibility, perceived severity, perceived benefits, perceived barriers, cues to action and self-efficacy. In our study, the HBM is particularly relevant because we are interested in understanding the beliefs of mothers regarding the severity of cervical cancer and the susceptibility of their sons to HPV, which are critical factors that influence vaccine acceptance. Additionally, we will investigate the potential barriers that could prevent mothers from vaccinating their sons, as well as the cues to action that could encourage them to do so.

This research is particularly significant because it will help to raise awareness about the importance of HPV vaccination for boys, which has been relatively under-studied in Africa, particularly in Ghana. By increasing awareness of the benefits of HPV vaccination for both males and females, we hope to improve the acceptance rate of the vaccine and encourage more parents to vaccinate their sons against HPV. Furthermore, our findings will be useful in informing policy decisions related to the cost of HPV vaccination, as well as other related healthcare services. By understanding the factors that influence vaccine uptake, targeted policies that address specific barriers to vaccine acceptance, reduce costs, and increase the overall uptake of HPV vaccination could be developed.

## Methods

### Research design

A qualitative exploratory design was employed to fully understand the participants in terms of their knowledge, acceptance, and attitudes toward HPV vaccination in their sons. A qualitative exploratory design is suitable to examine vaccination views and acceptance due to the novel research area and the potential to explore complex and nuanced aspects of individuals’ beliefs, attitudes, and behaviors related to vaccination. Employing this method is advantageous when investigating under-researched areas such as HPV vaccination in boys, as it enables researchers to gather rich data on the factors that affect vaccine acceptance or rejection among mothers by obtaining an in-depth understanding of participants’ experiences and perspectives.

.

#### Inclusion criteria

The current study was careful to include mothers who had male children between the ages of 9 to 12 years, with no age limits placed on such mothers.

#### Exclusion criteria

Participants exempted from the study were those, who were neither parents nor in the position of parenting, either through biological means or through adoption. Also, mothers with male children below 9 years and 12 years and above. Lastly, mothers who were unwilling to partake in the study.

#### Sampling technique

The study employed the use of purposive sampling techniques to select the participants of the study. It was opined that the rationale for this technique helps improve the trustworthiness of the data and results [26]. This is because it allows the selection of participants who met both the inclusion and exclusion criteria and were willing to partake in the study after an informed consent form was obtained from them. Purposive sampling was employed, and as such, only churches situated within the Shai Osudoku District, particularly Dodowa, which serves as the district capital, were chosen due to the representation of congregants from various other parts of the district, including Ayikuma (4), Doryumu (4), Manya-Jorpanya (3), Dodowa (12), Asabe (2), Asutsuare (3), Kodiabe (2), and Agomeda (4). The churches that were used in the study were Roman Catholic (15), Methodist (3), Pentecostal (5), Baptist (3), Anglican (2), and Seventh-day Adventist (SDA) (2), totaling six in number. The study was announced in the churches by the church leaders, and most of the mothers expressed interest. Nonetheless, the researchers selected mothers with male children between 9 and 12 years of age, who were attending churches situated within the Shai Osudoku District using purposive sampling.

#### Sample size

Estimating a qualitative sample size prior to conducting a study is an intrinsically challenging approach, which is particularly accentuated in more interpretive instances of qualitative research [27]. In the research process, saturation denotes the point at which data analysis no longer yields new findings, signalling to researchers that data collection can be terminated [28]. The researcher, therefore, interviewed participants till no new information had been obtained or retrieved. For this reason, the sample size for this was based on saturation, as it was the most preferred concept in qualitative research and was reached at 30, however, similar opinions were noted at the 27 participants, and all the other three participants were giving similar views, hence the data collection was terminated on the 30th participant.

#### Data collection procedure

Ethical clearance was obtained from the Dodowa Health Research Centre Institutional Review Board (DHRCIRB/145/11/21). Initially, an introduction and the ethical clearance form were submitted to the chosen district within the Greater Accra Region. The researchers also sought permission from the leaders of the churches located in the district. Upon receiving approval from the leaders of the selected churches, the researchers introduced themselves to the participants, elucidated the study’s objectives, and provided a clear explanation of the keywords used in the research instrument. Subsequently, a semi-structured interview guide prepared by the researchers was utilized. The guide had 3 sections that elicited questions on the socio-demographic characteristics of participants, the knowledge, and barriers to HPV vaccination in boys. The guide was pre-tested among 4 participants from two different churches in Accra which was not included in the main interviews.

After conducting a pilot study, face-to-face interviews were carried out in English at the selected churches for the study. The researchers used audio recorders during the interviews to document the participants’ views, paying adequate attention to avoid missing any vital information. The contact information of interested eligible participants was collected, and interviews were scheduled at a time and place that was convenient for them. The interviews were conducted in different settings, including the church outside of session times and the participants’ homes. To minimize disruptions or distractions, only the researchers and participants were present during the interviews. Mothers were selected for the interviews due to their significant influence over their children’s decision-making, particularly for those under 18 years of age. Moreover, the mothers were primarily responsible for their children’s healthcare needs and well-being.

At the time of the interview, participants who showed unwillingness were allowed to opt-out of the interview. Those who agreed to participate in the study were assigned pseudonyms in identification to enhance confidentiality. The researcher explained all questions in simple language to participants to answer correctly. The discussions lasted between fifty (50) to sixty (60) minutes. Data collection took place over a period of three months and strict adherence to COVID-19 safety protocols was observed. Social distancing was maintained during the interviews, and both researchers and participants wore face masks. A box containing face masks and hand sanitizer was readily available to ensure compliance with the protocols. Participants were thanked for their valuable contributions at the end of each interview.

#### Data analysis

Data analysis is defined as the process of cleaning, transforming, and modeling data to find useful information for decision-making [29]. Inductive thematic data analysis was used for this study which was guided by Health Belief Model Theoretical Framework. According to Braun and Clarke, thematic analysis ought to serve as a fundamental approach to qualitative analysis since it equips researchers with fundamental competencies that are crucial in executing a wide range of other forms of qualitative analysis [30]. Thematic analysis aids in summarizing large data sets by encouraging a structured approach to data handling, resulting in a clear and organized final report.

There are six stages in thematic analysis [31]. The six stages are as follows: Familiarization, generation of codes, searching for themes, reviewing themes, defining and naming themes, and producing report [30]. The themes were also organized with NVivo 12 software. In the first stage which is familiarization, the researchers got to know the data that has been collected by reading and rereading through the transcripts for a thorough understanding to aid in the coding of the data. See Table 1 for a detailed description of the thematic analysis.

**Table 1:** Description of thematic analysis

STAGES	MEANING	Applicability
Familiarization	The process by which researchers engage deeply with the transcribed data is known as immersion.	The audio recorded information was transcribed into a word document by the researchers, who then went through the data multiple times to become acquainted with it.
Coding	Coding refers to the act of labeling participants’ ideas with brief terms.	The researchers accomplished this by utilizing 2–3 word phrases that precisely conveyed the participants’ ideas across all 30 transcripts. For example some of the codes included “Vaccination centers” “Vaccination effectiveness” “Vaccination views” “Vaccination misconceptions”
Searching for Themes	At this stage, pertinent coded data extracts are categorized and consolidated into thematic units.	The authors collectively determined the thematic categories based on the theoretical framework, and grouped all similar codes under each theme. For example suitable theme for the above codes was “vaccination knowledge”
Reviewing Themes	This process involves the authors reviewing and refining the themes.	All authors participated in this process, which involved removing duplicate themes and collapsing some themes. They also verified that the themes aligned with the chosen theoretical framework. The two themes generated were. “Vaccination knowledge” and “Vaccination barriers”
Defining and Naming Themes	This phase entails the adjustment and refinement of theme definitions.	The authors engaged in peer debriefing and consulted with experts in the field to finalize the themes. They also made certain that the process was not isolated from the theoretical framework. The two themes were “Knowledge of mother on HPV vaccination” And “Barriers to HPV vaccination in boys”
Producing the Report	this is the ultimate stage that entails presenting the themes in a coherent manner	In the results section, the authors logically presented the themes, bolstered by verbatim quotations from the participants.

The second stage involves assigning codes to the data collected. This was done by labelling participants’ ideas with 2–3 words that had the same meaning as the participants’ words. The third stage involved the generation of themes, Comments, and expressions of close nature that were grouped together to form sub-themes. Finally, was reviewing the themes. The themes were examined and improved by collapsing some themes into other themes. This was to ensure that all the data followed a consistent pattern. For the purposes of this study, the developed codes were tabulated and arranged into main and sub-themes.

#### Methodological rigour

Methodological rigor is termed as the trustworthiness of a qualitative study [32]. The rigor is dependent on the four stringent dimension criteria developed by some Lincoln and Guba which are credibility, dependability, confirmability, and transferability. Credibility refers to confidence in how well the data addresses the intended focus of the study [33]. The researcher had the opportunity to speak with all willing parents across the churches for the data. The researcher made sure the data collection process and data collection were well planned. Dependability establishes the consistency and reliability of the research findings and confirmability is the extent to which the findings of the research study can be validated by other researcher [34]. To achieve this, the researcher provided a detailed description of the participant’s views on the issues and the research methodology.

Transferability of a research finding is the degree to which it can be applied in other parts and studies [35]. Purposive sampling technique was used to make sure that the selected participants were indeed the representation of the variety of respondents we faced. This was critical in conducting a well-informed analysis.

## Results

### Analysis of socio-demographic data

The socio-demographic characteristics examined from the 30 respondents included: age, marital status, number of children, educational status, occupation, and ethnicity. The age of participants ranged from the least age (25 years) to the highest which was 50 years. Half of the participants (50%) fell within the age group of 31 and 40 years, followed by the age range of 41years to 50 years with a percentage of 33.3 and the least age group was between 25years to 30 years having only 16.7% of the participants in that age category. Most of these participants (83%) were married with families, followed by 10% of them being single and 1% of the participants being divorced and widowed respectively. The rest of the data is presented in Table 2.

**Table 2:** Socio-demographic characteristics of respondents

**Variable**	**Frequency (n = 30)**	**Percentage (%)**
**Age group**		
25–30	5	16.7
31–40	15	50
41–50	10	33.3
**Marital status**		
Single	3	10
Married	25	83.3
Divorced	1	3.3
Widowed	1	3.3
**Number of children**		
1	4	13.3
2	11	36.7
≥ 3	15	50
**Educational Status**		
Primary	4	13.3
Secondary	8	26.7
Tertiary	18	60
**Occupation**		
Government workers	4	13.3
Private workers	13	43.3
Self-employed	12	40
Not working	1	3.3
**Tribe**		
Akan Ewe Ga Northerner Nigerians	11 9 7 1 2	36.6 30 23.3 3.3 6.7
**Churches**		
Roman Catholic Pentecost Methodist Anglican Seventh-Day Adventist Baptist	15 5 3 2 2 3	50 16 10 7 7 10

### Organization of the themes

Two primary themes and eleven sub-themes emerged from the data. The first theme focused on the mothers’ knowledge of HPV vaccination, while the second theme explored the barriers to HPV vaccination for boys.

The first theme, “Knowledge of mothers on HPV vaccination,“ had 5 sub-themes, which were views on HPV vaccination, mode of transmission, HPV vaccination centers, the effectiveness of HPV vaccination, and misconceptions about HPV vaccination. The subthemes categorized under the second theme (barriers) were cultural barriers, religious barriers, fear of pain from HPV vaccination, cost, side effects, fear of HPV vaccination making men immoral, and fear of HPV vaccination causing infertility in men.

### Theme 1: Knowledge of mothers on HPV vaccination

**Fig. 1:**
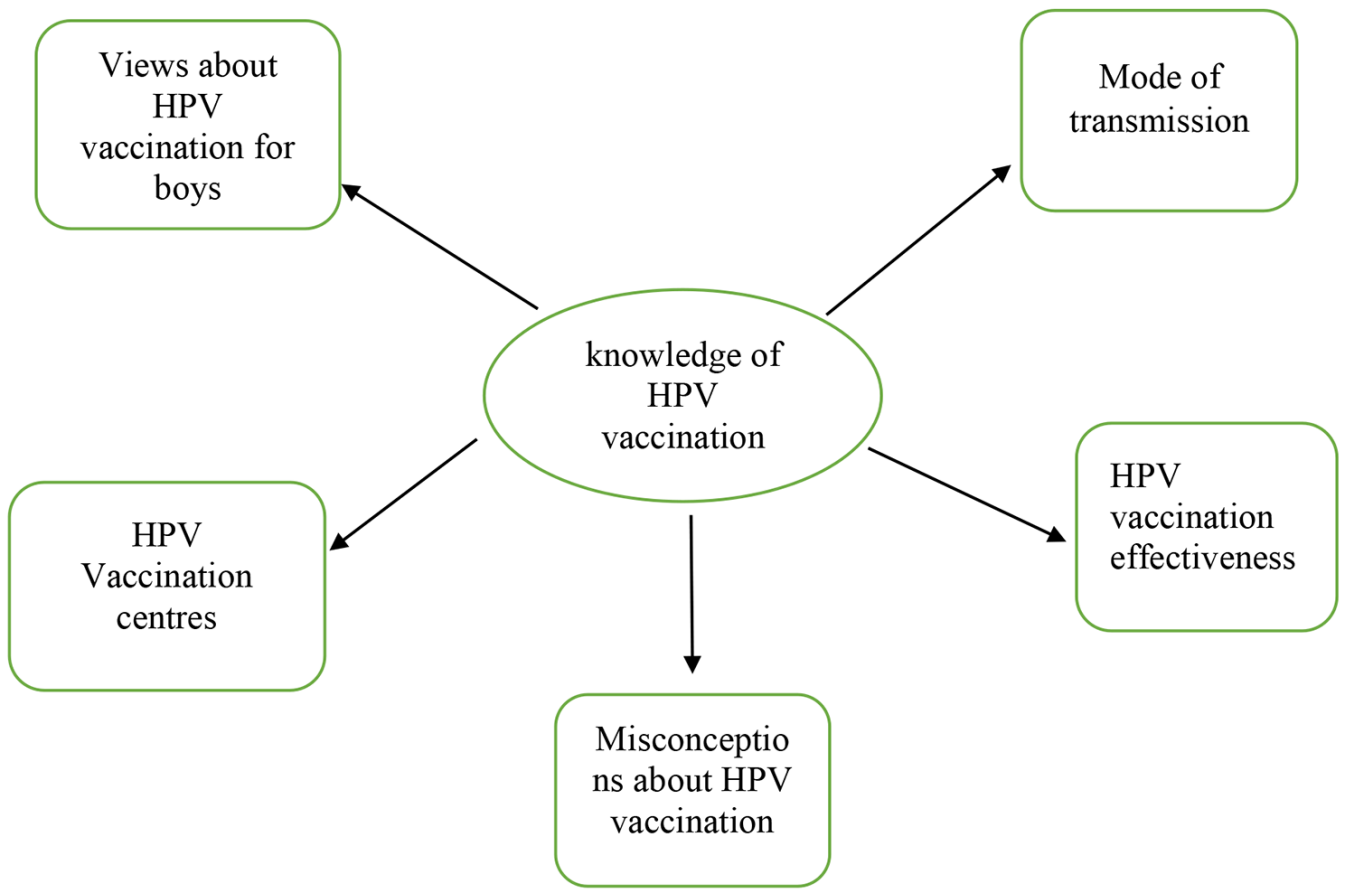
Theme 1: Knowledge of mothers on HPV vaccination and subthemes

Mothers’ knowledge about HPV vaccination varies widely. Some are well-informed and recognize its importance in preventing cervical cancer, while others have misconceptions about the vaccine’s safety and efficacy. The first theme includes five sub-themes that cover mothers’ views and knowledge of HPV vaccination, the mode of transmission, vaccination centers, vaccine effectiveness, and misconceptions surrounding the vaccine. See Fig. 1.

#### Views on HPV vaccination for boys

The research revealed that mothers held varying opinions on giving their young sons the HPV vaccine. Certain mothers believed that their sons were too young to receive the vaccine, despite its benefits in preventing HPV, while others had no reservations about vaccinating their male children.

*“The HPV vaccination in boys is a good thing since I know all vaccines protect against a particular disease. So for me, I want my son to wait since he is too young (9 years) till he is 18 years before I will send him to be vaccinated”*** N13**

“Every day you people bring us something new. *I don’t really know about this vaccine you are talking about, but if there is a vaccine like that, I think it is a good idea and I do not know why I should not let my sons go for it”* N2.

Below are responses of participants who mentioned that they were not aware of HPV vaccination and believed that their children have already been vaccinated and hence there is no need for further vaccination

*“This HPV vaccination is new to me, honestly this is my first time hearing of it, and besides my twin boys have already been immunized against TB, Measles, and others, so why should they now take the HPV vaccine again? I think they can all protect him against this virus you are talking about.”*** N4.**

*The HPV is not an African thing is only the whites who get it. I don’t personally know anyone nor have I heard of anyone who has it so why must my child take it?.This sickness is for the white people just like the covid-19. They should leave us alone”*** N11**.

#### Mode of HPV transmission

Some held the belief that HPV vaccination was unnecessary as it is sexually transmitted, and their sons were not sexually active.

*“What I know about this HPV is that if you don’t have sex then you are less likely to contract it and also unlikely to transmit it to another person. Per this understanding, I don’t think my son has to take the vaccine since he is not sexually active, and you can only contract it through sex”*** N7**

Few participants in this study were unaware of the mode of transmission for HPV

*“My boy plays with a lot of children in my vicinity, so does this mean he is at risk of contracting the virus from them? Besides, when the health talk was given at church, I remember the lady saying it affects women so we should be concerned about our girls. What has this got to do with my boy? …”*** N20**.

#### HPV vaccination centers

The study found that the majority of mothers were unaware of where the vaccination would be administered, and those residing in rural areas believed that accessibility would be a challenge even if the vaccination were introduced.

*“My concern is where to locate such places if indeed vaccine will be made available. You know Ghana, sometimes you go to public hospitals and you don’t get all the services you need. You must move to another place before you can do a certain test requested. It makes it stressful; that’s why if they are going to do this vaccination then it should in at least every district hospital. This way I know that when I go to this facility I’ll get the vaccine and not be roaming”*** N17**

*“This is a challenge for those of us staying in the rural areas, we only have access to health centers, and these screenings are not done there so it is even a problem where to get it if we agree to vaccinate our boys.”*** N8**

*“I have heard that some women go for Cervical Cancer screening at the KorleBu Teaching Hospital and also take the vaccine there, but I don’t know if boys can also get the HPV vaccination at the same place”*** N19**

#### HPV vaccination effectiveness

Their concerns varied, with some questioning the effectiveness of the vaccine, as they feared a newer, more potent version may be introduced after its initial release. Additionally, they worried that their children may still contract HPV even after receiving the vaccine.:

*“My concern is not allowing my boys to take the vaccine, but I am worried about the fact that after they take it, they might introduce another vaccine just like Covid 19 as a booster”.*** N14**

*“I don’t think it will be 100% effective, because, I took a Covid 19 vaccine, and after I was still diagnosed of Covid 19 so I don’t believe any vaccine gives 100% protection.”****N9***

Some believed that their perception of the vaccine’s effectiveness was influenced by the testimonials provided by healthcare providers as follows: *“If the doctors and the nurses are saying this vaccine works then I’ll believe you. After all, it is your field and you know what you’re doing. Just like the immunizations you give our children you know what you are doing and also tell us why you are doing it so I know it’s for the good of my children. So it is the same here too”*** N22**

#### Misconception about HPV vaccination

Mothers held various misconceptions about the vaccine, including the false belief that it contained a tracking microchip, denial of the existence of cervical cancer, mistrust in the vaccine, and suspicion that it was a means of extorting money

“*These new vaccines that are been developed currently like the Covid 19 vaccine, I hear have microchips in them that can help in tracking, monitoring and also controlling human beings. So I didn’t take the covid vaccine how much more allowing my son to take this HPV vaccine. I honestly don’t believe that this HPV vaccine will protect my son”.****N18***

*“I don’t believe there is any disease like that…you people just want to introduce some disease into our children then later come back and tell us you have a cure. It is just a means for you health workers to extort money from us…as for me, I won’t vaccinate my son, you people say this, tomorrow that I’m tired of you people”*** N27**.

### Theme 2: Barriers to HPV vaccination among boys

**Fig. 2:**
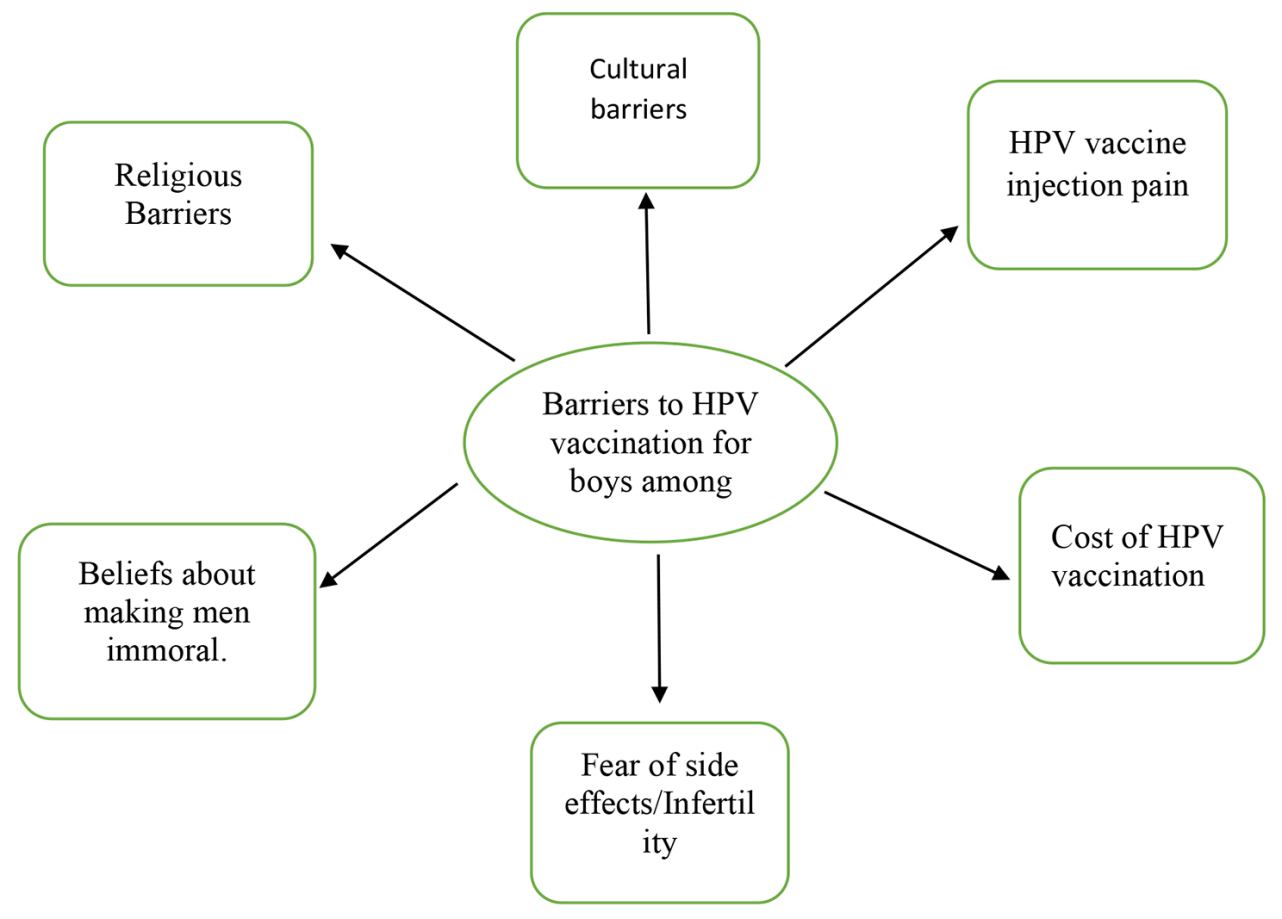
Theme 2: Barriers to HPV vaccination among boys and subthemes

This refers to the challenges and obstacles that prevent boys from receiving the HPV vaccine, such as social stigma, lack of awareness, safety concerns, and cost issues. Addressing these barriers is important to improve vaccination rates. The subthemes included cultural and religious factors, fear of pain and side effects, concerns about cost, and fears of perceived negative impacts on morality and fertility. See Fig. 2.

#### Cultural barriers

The majority of mothers in this study believed that culture had little influence on vaccinating children, as indicated below.

*“ I am Fante, my tribe and culture do not have any issues with young boys being vaccinated, once it’s for a good course then they have no issues. But come to think of it, in this 21st century, do people still allow their culture to influence their decision when it comes to health(laughs). Well, my culture has no issues with this. Even if it has, life is more important to me.*** N29**

*“Culture or no culture I am all for this HPV vaccination, I am from a royal family, we rely greatly on herbal medicine. It was in the olden days that orthodox medicine wasn’t trusted but now, because of exposure it has been accepted. So my culture should not prevent me from getting my son vaccinated”****N26***.

On the other hand, some mothers expressed their concerns and admitted to the fact their culture is a contributory factor to their vaccine hesitancy.

*“I come from the Northern part of Nigeria, and we don’t joke with our male children, especially the first born, any form of needle, sharp object that will cause pain we are really hesitant about it, so this HPV that I know little about, I won’t expose my son, no harm must come to him”*** N23**.

#### Religious barriers

Most mothers also expressed their belief that they need not worry about vaccines since they have faith in God and believe that they and their children are fully protected.

“Ideally *if my son follows Christ and is obedient, then there is no need for him to take the vaccine because the disease is sexually transmitted. As a Christian, you have no business fornicating, so he doesn’t need the vaccine. I would rather nurture him the fear of the Lord to prevent this HPV virus”*** N1**.

*“I have read in the Bible where a lot of good standing Christians received their healings, so I believe God will protect my children from all kinds of diseases if I trust in him” N5*

#### Mother’s fear regarding HPV injection pain

One of the major concerns expressed by mothers in this study was the fear of their children experiencing pain following vaccination, which made them hesitant to allow their children to receive the vaccine.

*“I am a little worried about the pain my son will experience after taking the vaccine. This vaccine is new to me and I don’t how he will feel after the vaccine. Even with the TB vaccine and others, my son was in pain for quite some time, it wasn’t easy for me at all”.*** N2**

*“They are kids, I hope it won’t be too painful, even with me when I took the Covid-19 vaccine I could barely lift my arm for like 3 days, when my boy start crying, you can’t control him.*** N20**

Few of the participants were unconcerned about the pains that will be experienced by their boys resulting from the HPV vaccine.

*“Well, I am not too bothered about my son experiencing pain, because it is expected. I just have to deal with the pain after he takes the vaccine. Injections always come with pain and you know it.”*** N25**

#### Cost of HPV vaccination

The cost of the HPV vaccine was also a major concern expressed by the mothers, who suggested that the vaccine should be made available at a lower or no cost for them to allow their children to receive it.

*“I hope the HPV vaccine will be for free because most of the vaccines that our children receive when they are born are for free. The government can also include it in the National Health Insurance Scheme, especially for those in the villages.”*** N21**

*“Honestly this vaccine is not compulsory, but it is in the best interest of our sons, if you the health workers start the vaccination exercise and it is expensive, I won’t bother myself. So please make sure that the vaccine is affordable if not forget it (laughs)”*** N16**

Few were unconcerned about the cost of the HPV vaccine.

*“My son’s wellbeing is my top most priority, so I am not looking at the cost. If the HPV vaccine will protect him why not, I am willing to pay any amount. God forbid but he dies right now because of this HPV, I can’t resurrect him. So prevention is better than cure”*** N11**

#### Belief about making men immoral

Some participants in the study believed that administering the HPV vaccine to their young boys could create a false sense of security and lead them to engage in sexual activities without the fear of contracting the virus. They were concerned that this could potentially lead to negative consequences.

*“Allowing my son to take HPV vaccination is like telling him that he is free to sleep around because he is protected. Children in this 21st Century are always looking for opportunities to justify the wrong things they do. Can you imagine the kind of things they would get themselves into knowing that they have been vaccinated against a sexual infection?”*** N24**

*“I know the purpose of this vaccine is to protect our young boys, but indirectly it is going to promote sexual promiscuity, what worries me the most is that they are young and very curious at this stage, I am just wondering the kind of things they will be doing after taking a vaccine like this.”*** N11**

#### Fear of HPV vaccination side effects (infertility in men)

Fear of potential side effects is one of the main reasons for vaccine hesitancy. Infertility in their young boys stood out as their greatest fear with regard to the side effects especially because it was new to them.

*I stand to be corrected but I think this vaccine can affect my sons’ ability to have kids, this vaccine was created for sexual infection. People react to medications in different ways what if my son’s reaction leads to infertility, what will I do?*** N19**

*I don’t want to get myself into this HPV vaccination and regret it later. If you can show people who have taken the vaccine and have kids, then I will be fully convinced*** N4**

On other the hand, some mothers were convinced that the HPV vaccine would not cause serious side effects to their boys.

*“I don’t think my son will become infertile from taking the vaccine, I believe the vaccine would been tested several times before being given to humans, so I doubt it would cause infertility.”*** N22**

## Discussions

The study revealed insufficient knowledge of HPV vaccination among mothers, however, they perceive the vaccine to be of immense benefit on the basis that vaccines help protect children from disease. Due to this, the mothers recommended the need for healthcare professionals to increase information on HPV vaccination to help increase the acceptance rate. This implies that mothers will be more willing to send their male children for vaccination if they are told more about the vaccine and its benefits. Similarly, a study revealed that most fathers and mothers showed poor knowledge of the vaccination which can be attributed to their decision to allow their young boys and girls to take the vaccine [36]. The HBM model was applicable in this study, as it revealed a link between increased knowledge and behavior outcomes. This suggests that promoting awareness of HPV vaccination for boys could increase uptake and acceptance.

The present study revealed that most participants exhibited a sound understanding of the mode of transmission of the HPV virus, recognizing it as a sexually transmitted infection. This understanding led some participants to perceive HPV as more prevalent among females than males. However, some participants believed that safeguarding their sons from premarital sex could provide protection against the virus, given its sexual transmission. These attitudes underscore the need to enhance public awareness about HPV, particularly among those who believe it to be a disease that primarily affects women. Similarly, a study found that a mere quarter of participants 34.3% were aware that HPV is a sexually transmitted infection, and only 40.4% were aware of its association with cervical cancer [37]. These findings emphasize the pressing need to implement targeted health education programs to enhance public awareness about the risks posed by HPV and its links to cancer. Considering these results, efforts should be made to design effective health education initiatives aimed at raising awareness about HPV among the general public, with an emphasis on combatting misconceptions and addressing knowledge gaps. Such efforts can play a crucial role in empowering individuals to make informed decisions about their sexual health and ultimately contribute to the prevention and management of this significant public health issue.

In the current study, participants raised concerns regarding the availability of HPV vaccination centers in Ghana. Specifically, they noted that cervical cancer screening and vaccination centers were not widely available in hospitals across the country, leading them to believe that the situation would be similar for HPV vaccination in boys. Participants emphasized that their willingness to vaccinate their sons would depend on the availability and accessibility of the vaccine and associated vaccination centers. In light of these concerns, participants recommended that the vaccine be made widely available in clinics across the country to ensure ease of access. Interestingly, a study conducted by Rezqalla et al. in Kuwait found that a significant majority of female school teachers (88%) were unaware of the availability of the HPV vaccine [38]. This highlights the importance of not only making the vaccine available but also ensuring that the public is well-informed about its availability and the benefits of vaccination [21].

The results of the present study shed light on the perceptions of mothers regarding the effectiveness of the HPV vaccine. The doubts expressed by these mothers were largely influenced by their previous experiences with the COVID-19 vaccine, which caused them to question the ability of any vaccine to offer 100% protection. As such, it is crucial for healthcare workers to provide comprehensive education on vaccines, including their mechanisms of action and potential benefits, to alleviate the concerns of these mothers. This, in turn, could foster greater trust in the HPV vaccine and increase the likelihood of their willingness to vaccinate their sons. Notably, a majority of the parents in this study voiced concerns about the safety and efficacy of the HPV vaccine [39].

Most of the participants in the study had an accurate understanding of the HPV vaccination and welcomed its implementation for boys as a positive development. They recognized that this would not only benefit their sons but also contribute to the prevention of cervical cancer in their daughters. As a result, the more informed the participants were about the vaccine, the more likely mothers were to support the idea of vaccinating their young boys against HPV. It is noteworthy that a significant proportion of participants in another study held misconceptions about vaccines in general, with around 85% underestimating the magnitude and scope of clinical trials conducted for vaccines [40]. The findings are supported by the perceived benefits of the health belief model, which suggests that individuals are more likely to engage in health-promoting behaviors if they believe they are beneficial. Due to the high perceived benefits of the HPV vaccine, the majority of mothers in the study were willing to permit their young boys to receive it.

The study found that most of the participants who were interviewed did not encounter cultural obstacles when it came to vaccinating their young sons. Some participants believed that cultural beliefs should not impede individuals from making informed decisions about their health, particularly in the modern era. These mothers should be encouraged to discuss the advantages of the HPV vaccine with other mothers and encourage them to consider having their sons vaccinated. Notably, Ng and Tan’s research revealed a negative correlation between cultural rigidity and the inclination to receive vaccines [41].This finding is also in line with the assumptions of the HBM, which suggests that people’s behavior is influenced by external factors such as cultural, social, and environmental factors, as well as the opinions of relatives and health professionals.

The study’s findings showed that most of the participants did not encounter any conflict between their religious beliefs and vaccinating their sons against HPV. They held the belief that it was God who granted health professionals the knowledge and skills to develop vaccines, and thus had no reservations about having their sons vaccinated. These mothers could collaborate with spiritual leaders to promote the benefits of HPV vaccination to other mothers. Other researchers has demonstrated that religious convictions can have an impact on health-related decisions [42]. .

The study’s findings showed that, among the potential side effects identified, the possibility of infertility in their sons was the most significant concern for some mothers. They feared that the HPV vaccine could have a detrimental impact on their sons’ reproductive systems, particularly since the vaccine protects against a sexually transmitted infection. Other participants requested evidence to reassure them that the vaccine did not impair male fertility before trusting the HPV vaccine. To allay these fears, it is crucial to provide such mothers with evidence of the safety of the HPV vaccine. It is noteworthy that a survey demonstrated no evidence of increased infertility in women who received the HPV vaccine [43].

Some mothers expressed concern over the potential for pain associated with the HPV vaccination. Their apprehension stemmed from prior experiences with childhood vaccinations or the Covid-19 vaccine. They found it stressful and upsetting to see their children in pain and were hesitant to have them receive the HPV vaccine. It is crucial to provide these mothers with information on strategies they can use to help their children manage any discomfort and to reassure them that the pain is short-lived. Baxter et al. identified fear of needles as a barrier to the uptake of the HPV vaccine, while other barriers to cervical cancer screening and vaccination in Ghana included the high cost and doubts about its effectiveness [23, 44].

### Recommendations and implications to practice, future researchers and policymakers

For practice, the study highlights the importance of targeted public health education campaigns to increase knowledge and awareness about the HPV vaccine and its benefits. It is important for healthcare providers to be able to effectively communicate with parents about the importance of HPV vaccination and address any misconceptions or concerns they may have. The study also underscores the importance of making the HPV vaccine accessible and affordable for all families. For future researchers, this study provides important insights into the specific barriers to HPV vaccination in boys and the factors that influence mothers’ decision-making. The study highlights the need for further research to identify effective interventions to address these barriers and improve HPV vaccination rates in boys. Policymakers should also consider ways to address the specific barriers to HPV vaccination in boys, such as concerns about pain or fertility, in order to increase vaccination rates and reduce the burden of HPV-related diseases.

## Conclusion

In conclusion, the study highlights a lack of awareness among mothers regarding the HPV vaccine for boys. To increase its acceptance, there is a need to increase awareness of the vaccine not only among mothers but also among the public. Such efforts could help to dispel misconceptions, address concerns, and increase the uptake of the vaccine among boys.

## Data Availability

All data generated or analyzed during this study have been uploaded with this article.
